# Supplementary material for: Child marriage and its association with morbidity and mortality of under-5 years old children in Bangladesh
Source: PLoS One. 2022 Feb 9;17(2):e0262927. doi: 10.1371/journal.pone.0262927 (PMC8827428; doi:10.1371/journal.pone.0262927)
Supplement: S1 Table — (DOCX) [file pone.0262927.s001.docx]

**S1 Table:** Prevalence of Child Morbidity and Mortality Indicators by Socio-economic and Demographic Characteristics, BDHS-2017/18

| **Socio-economic and demographic characteristic** | **Labels** | **Morbidity indicators (%)** | | | **Mortality indicators (%)** | | | |
| --- | --- | --- | --- | --- | --- | --- | --- | --- |
|  |  | **Diarrhea** | **Fever** | **Cough** | **Under-5** | **Infant** | **Neonatal** | **Post-neonatal** |
| Age of child (in months) | 0-11 | 5.46 | 36.04 | 36.99 | - | - | - | - |
|  | 12-23 | 8.99 | 40.36 | 41.25 | - | - | - | - |
|  | 24-35 | 5.02 | 32.97 | 37.81 | - | - | - | - |
|  | 36-47 | 2.45 | 29.30 | 32.56 | - | - | - | - |
|  | 48-59 | 1.48 | 26.82 | 30.92 | - | - | - | - |
| Sex of child | Male | 5.06 | 34.23 | 37.64 | 12.32 | 80.51 | 64.06 | 16.41 |
|  | Female | 4.32 | 31.80 | 33.75 | 16.47 | 75.21 | 57.17 | 18.03 |
| Place of residence | Urban | 4.42 | 30.95 | 34.76 | 13.66 | 79.52 | 60.35 | 19.16 |
|  | Rural | 4.79 | 33.86 | 36.16 | 14.35 | 77.70 | 61.15 | 16.55 |
| Religion | Muslim | 4.87 | 33.55 | 35.86 | 14.51 | 77.61 | 60.11 | 17.50 |
|  | Non-Muslim | 2.83 | 27.53 | 34.82 | 11.08 | 83.10 | 69.53 | 13.57 |
| Place of delivery | With Health Facility | 6.70 | 35.14 | 39.77 | 1.00 | 99.00 | 87.00 | 12.00 |
|  | Respondent's Home | 6.27 | 37.63 | 37.32 | 6.98 | 93.02 | 81.18 | 11.76 |
| Currently breastfeeding | No | 2.70 | 30.39 | 33.93 | 14.26 | 77.45 | 59.88 | 17.59 |
|  | Yes | 6.11 | 34.95 | 37.07 | 13.73 | 83.19 | 69.28 | 13.76 |
| Delivery by C-section | No | 6.49 | 36.69 | 37.86 | 3.97 | 96.03 | 84.67 | 11.92 |
|  | Yes | 6.43 | 35.86 | 39.84 | 2.86 | 97.14 | 82.86 | 13.89 |
| Father's education | No education | 3.72 | 32.17 | 35.22 | 15.74 | 75.51 | 57.80 | 17.70 |
|  | Primary | 5.49 | 33.06 | 36.49 | 13.01 | 80.17 | 62.02 | 18.13 |
|  | Secondary and above | 4.54 | 33.28 | 35.37 | 12.29 | 80.37 | 65.84 | 14.46 |
| Mother's Education | No education | 6.22 | 30.11 | 29.41 | 17.54 | 73.10 | 54.23 | 18.87 |
|  | Primary | 4.53 | 33.67 | 35.23 | 12.98 | 79.54 | 62.63 | 16.91 |
|  | Secondary and above | 4.59 | 33.13 | 36.74 | 11.27 | 83.14 | 68.15 | 14.99 |
| Mother's BMI | Thin (<18.5) | 5.93 | 34.61 | 38.19 | 14.59 | 79.54 | 61.74 | 17.76 |
|  | Normal (18.5–24.9) | 4.57 | 33.86 | 35.61 | 14.98 | 77.49 | 60.77 | 16.72 |
|  | Obese (25 and more) | 4.44 | 31.21 | 35.54 | 12.35 | 78.78 | 61.09 | 17.64 |
| Division | Barisal | 6.47 | 38.36 | 37.72 | 19.84 | 68.83 | 49.39 | 19.43 |
|  | Chittagong | 5.05 | 32.48 | 33.05 | 18.50 | 73.86 | 56.68 | 17.18 |
|  | Dhaka | 3.82 | 30.74 | 32.21 | 13.28 | 80.35 | 62.64 | 17.69 |
|  | Khulna | 3.88 | 31.27 | 36.05 | 9.32 | 81.37 | 63.56 | 17.81 |
|  | Mymensingh | 5.37 | 33.33 | 37.57 | 12.46 | 80.45 | 62.32 | 18.13 |
|  | Rajshahi | 5.73 | 34.60 | 41.15 | 15.65 | 77.34 | 63.10 | 14.14 |
|  | Rangpur | 4.41 | 36.27 | 41.92 | 11.11 | 79.17 | 64.48 | 14.71 |
|  | Sylhet | 4.55 | 33.82 | 34.99 | 12.89 | 81.45 | 60.38 | 21.07 |
| Wealth index | Poorest | 4.91 | 34.19 | 37.22 | 15.53 | 77.24 | 59.21 | 18.03 |
|  | Poorer | 4.62 | 33.27 | 36.43 | 15.05 | 76.34 | 58.36 | 17.98 |
|  | Middle | 5.92 | 34.53 | 35.98 | 15.32 | 77.01 | 61.95 | 15.08 |
|  | Richer | 3.31 | 35.13 | 35.64 | 11.04 | 81.04 | 64.85 | 16.08 |
|  | Richest | 4.81 | 28.11 | 33.46 | 12.63 | 80.84 | 62.32 | 18.53 |
