# Supplementary material for: Child marriage and its association with morbidity and mortality of under-5 years old children in Bangladesh
Source: PLoS One. 2022 Feb 9;17(2):e0262927. doi: 10.1371/journal.pone.0262927 (PMC8827428; doi:10.1371/journal.pone.0262927)
Supplement: S2 Table — (DOCX) [file pone.0262927.s002.docx]

**S2 Table:** Results of Logistic Regression, BDHS-2017/18

|  | **Variable** | | **B** | **S.E.** | **Wald** | **df** | **p-value** | **OR** | **95% CI o**f **OR** | |
| --- | --- | --- | --- | --- | --- | --- | --- | --- | --- | --- |
|  |  |  |  |  |  |  |  |  | **Lower** | **Upper** |
| **COR** | | | | | | | | | | |
| **Morbidity Indicators** | Diarrhea | Adult marriage | -0.04 | 0.02 | 4.33 | 1.00 | 0.04 | 0.97 | 0.97 | 1.18 |
|  | Fever | Adult marriage | 0.12 | 0.25 | 0.22 | 1.00 | 0.64 | 1.12 | 1.03 | 1.23 |
|  | Cough | Adult marriage | -0.15 | 0.06 | 5.54 | 1.00 | 0.02 | 0.86 | 0.72 | 1.16 |
| **Mortality indicators** | Under-5 mortality | Adult marriage | -0.08 | 0.04 | 5.18 | 1.00 | 0.02 | 0.92 | 0.84 | 1.33 |
|  | Infant mortality | Adult marriage | -0.20 | 0.08 | 5.87 | 1.00 | 0.02 | 0.82 | 0.74 | 1.02 |
|  | Neonatal mortality | Adult marriage | -0.25 | 0.07 | 13.46 | 1.00 | <0.001 | 0.78 | 0.68 | 0.89 |
|  | Post neonatal mortality | Adult marriage | 0.12 | 0.04 | 7.36 | 1.00 | 0.01 | 1.12 | 1.08 | 1.53 |
| **AOR** | | | | | | | | | | |
| **Morbidity Indicators** | Diarrhea | Adult marriage | -0.07 | 0.03 | 5.06 | 1.00 | 0.02 | 0.93 | 0.76 | 1.16 |
|  | Fever | Adult marriage | 0.05 | 0.02 | 4.43 | 1.00 | 0.04 | 1.05 | 1.01 | 1.22 |
|  | Cough | Adult marriage | -0.09 | 0.04 | 4.93 | 1.00 | 0.03 | 0.91 | 0.78 | 1.17 |
| **Mortality indicators** | Under-5 mortality | Adult marriage | -0.24 | 0.08 | 7.94 | 1.00 | 0.005 | 0.79 | 0.62 | 1.02 |
|  | Infant mortality | Adult marriage | -0.12 | 0.06 | 4.00 | 1.00 | 0.05 | 0.89 | 0.67 | 1.13 |
|  | Neonatal mortality | Adult marriage | -0.17 | 0.06 | 8.99 | 1.00 | 0.003 | 0.84 | 0.72 | 0.97 |
|  | Post neonatal mortality | Adult marriage | 0.12 | 0.04 | 8.55 | 1.00 | 0.003 | 1.13 | 0.86 | 1.20 |
