# Supplementary material for: Child marriage and its association with morbidity and mortality of under-5 years old children in Bangladesh
Source: PLoS One. 2022 Feb 9;17(2):e0262927. doi: 10.1371/journal.pone.0262927 (PMC8827428; doi:10.1371/journal.pone.0262927)
Supplement: Attachment — Submitted filename: Response to Reviewers.pdf [file pone.0262927.s004.pdf]

Author responses to the review comments:

We sincerely thank the Academic Editor and four anonymous reviewers for their valuable comments. We have considered all comments and then thoroughly revised and formatted the manuscript. A detailed response to each comment is provided in the following table.

**Editor/Academic Editor comments:**	**Response**	**Note**
Considering my own reading of the paper and all four reviewers observations, I am going with a decision of Reject and Resubmit.<br>Please submit your revised manuscript by Jan 09 2022 11:59PM. If you will need more time than this to complete your revisions, please reply to this message or contact the journal office at plosone@plos.org. When you're ready to submit your revision, log on to https://www.editorialmanager.com/pone/ and select the 'Submissions Needing Revision' folder to locate your manuscript file.<br>Please include the following items when submitting your revised manuscript:<br>A rebuttal letter that responds to each point raised by the academic editor and reviewer(s). You should upload this letter as a separate file labeled 'Response to Reviewers'.<br>A marked-up copy of your manuscript that highlights changes made to the original version. You should upload this as a separate file labeled 'Revised Manuscript with Track Changes'.<br>An unmarked version of your revised paper without tracked changes. You should upload this as a separate file labeled 'Manuscript'.	Thank you very much for your valuable comment and feedback. We believe that it will be helpful to improve the quality of the manuscript.<br><br>All necessary files are uploaded in the journal system.	All revised texts are in "red" color.
**Reviewer 1 comments:**	**Response**	**Note**
I have some minor suggestions. Please find them below:<br><br>1. Line 109- replace 'published BDHS datasets' with some other relevant word. 'Published' is not a correct word here.	Thank you very much for your valuable comment and suggestions that help improve the manuscript's quality.<br>We replace "published BDHS datasets" by "available BDHS datasets".	All revised texts are in "red" color.<br><br>Page: 4
2. In table 1 (and wherever relevant): place 'in month' in bracket after age of child. It should be- age of child (In months). Table 1 first demographic characteristic.	Thank you very much. Table 1 is revised as per your comments.	Revised texts are in red color.<br>Page: 7-8
3. Please confirm the p-value for teh sex of the child in table 1.	Thanks. Actually, we missed 0. It was typo. It would be 0.082.	The revised texts are in red color.<br>Page: 8
4. Authors may provide CIs for crosstabs also. This would increase the robustness of the study findings.	Thanks. We provide the CIs in Table 3.	Page: 11

5. Consider changing post-natal mortality to post neonatal mortality.	Thank you very much. We replace "post-natal" by "post neonatal".	The revised texts are in red color.<br>Page: 10-14
6. Furthermore, women who particularly lived in rural areas, are not permitted to visit a health care facilities for their own as well as their children's health [54,55]. In above sentence, authors cited quite old references. Either update the reference or consider omitting the sentence as the current situation may be different.	Thanks. The manuscript is revised as per guidelines.	The revised texts are in red color.<br>Page: 12-14
7. According to the findings of the study, illiterate and undereducated mothers and fathers married in their early age. In the sentence, consider replacing 'mothers and fathers' as when they married off, they were men and women and not mothers and fathers.	Thanks. We modify this sentence.	Revised texts are in red color.<br>Page: 14
**Reviewer 2 comments:**	**Response**	**Note**
Thank you for the chance to review the paper. It is an interesting paper that focuses on Child marriage and its association with child morbidity and mortality pattern in Bangladesh. Overall, the paper has explored a vital area; however, for strengthening the paper, here I have outlined my concerns and suggestions.<br>• Writing "early married women" Instead of "child married women" sounds more appropriate. I would suggest to rewrite using the suggested terms. (Found in lines – 36, 184, 213, 214, 217, 219, 223)	Thank you very much for carefully checking the manuscript and your feedback. It will help to improve the quality of the manuscript.<br>"child married women" is replaced by "early married women" in the revised version of the manuscript.	Revised texts are in red color.<br>Page: 1, 7, 9, 10
• The sentence "Girls who get married are often under pressure to give birth to a child early"- in line number 59 needs reference.	Thanks. We add the reference.	Revised texts are in red color.<br>Page: 2
• The statement in lines 81-84 consists factual information. Please provide reference supporting the information.	Thank you very much. We add references in the revised manuscript.	Revised texts are in red color.<br>Page: 3
• The statement in lines 270-271 mention about "map", however the map has been removed by the authors. Please modify the statement.	Thanks for highlighted this point. Yes, we remove the map from the revised version. Now, we modify this sentence.	Revised texts are in red color.<br>Page: 12
• The authors are suggested to incorporate "Child's BMI" and "Child's Immunization status" as controlled variable in the binary logistic regression model, since these variables can affect the morbidity and mortality of children under age 5. All these variables can be created using BDHS dataset.	Thanks. We think that it would be potential to include the child's BMI, immunization status in future study.	Revised texts are in red color.<br>Page: 14
• Reading discussion of this, I have found that most of the findings are found consistent with previous studies. Then the authors should clarify the contribution of this study to academia. More focus should be given to the unique findings first and then others.	We appreciate your feedback. We revised the discussion section.	Revised texts are in red color.<br>Page: 12-14

• Moreover, I would suggest to use PSM technique rather than logistic regression. The PSM method can strengthen the analysis and can stand as more appropriate for this study.	Thanks for your suggesting to use PSM technique. However, we think this would be a potential topic for future research. We add this in the limitation section.	Revised texts are in red color.<br>Page: 14
**Reviewer 3 comments:**	**Response**	**Note**
The central theme and the issue discussed here in the paper is significant and holds relevance to the country since as stated by the authors, in Bangladesh, 59% and 22% of women get married before the age of 18 and 15 years, respectively. And with about 31% of Bangladeshi girls becoming mothers before they are 18 years, this is a serious issue and should be studied thoroughly, As such, the analysis is timely and of potential value for policymakers. Appreciate the authors for taking such a relevant issue to study. This would definitely lead to an increase in the knowledge base in this domain. However, some problems with the writing of this paper should be addressed before the manuscript is accepted for publication.	Thank you very much for your appreciation and insightful feedback. We believe that your comments help to improve the quality of the manuscript.	
There are some more revisions required in writing. Some of the errors which might need revision are-<br>Line 32 Was declined- was declining<br>Line 33- is still high- was still high<br>Line 47- that affect- which in turn affect<br>Line 132- the authors take permission- the authors took permission<br>Line 189- urban areas, women who are from lower-class families get married at an early age.<br>Line 198 - categories was declined- categories declined<br>Line 199- mortality was declined- mortality declined<br>Line 204- was decreased- decreased<br>Line 215-216- moreover has been used repeatedly. The sentence should be modified.<br>Line 353- According to the findings of the study, illiterate and undereducated mothers and fathers married at their early age. -Rephrase the sentence.	Thanks for carefully checking the manuscript. We incorporate all your suggestions in the revised version of the manuscript.<br><br>We remove the sentence in line 188-189 because of repetition.	Revised texts are in red color.<br>Page: 1-2, 5, 7, 9-10, 14
Overall, English write-up needs to be checked carefully in the whole manuscript, sometimes some errors are overlooked by software. There should be a flow in the usage of tenses. The methodology is fine and easy to understand but it is basic, the authors could have used some more sophisticated methodology to compare and get a clear comparison picture of the two groups studies. Some Matching techniques can be used which would make the study unique and completely different from the already done work. Further, such a methodology would also help in understanding a few new dynamics of the factors contributing to child marriage and child mortality in Bangladesh.	We appreciate your feedbacks. The whole manuscript is checked carefully and fixed the grammatical issues.<br>We think that matching techniques will be incorporated in the future study. We add it in the limitation section.	Revised texts are in red color.<br>Page: 1-15

Incorporating these few changes, this study would be a valuable resource for future studies in this field.		
**Reviewer 4 comments:**	**Response**	**Note**
This article is based on cross-sectional data from a cross-sectional population survey with 8321 children under the age of five in Bangladesh. The first aim was to estimate the births to women born into child married and adult married women in Bangladesh by socioeconomic characteristics, defined as women age at first birth <18 years and >18 years. The second objective relates to the prevalence of morbidities and mortalities under 5 years' children by child marriage and adult marriage women. The author looks at three morbidities in the last two weeks, such as diarrhea, fever, and cough, as well as four mortality indicators, including infant mortality, neonatal mortality, and post-neonatal mortality. The findings revealed that child mortality among adult married women was considerably lower. However, there are certainly major issues that must be addressed.	Thank you very much for your comments and feedback. We revised the manuscript as per your comments.	Revised texts are in red color.
Introduction<br>Unfortunately, the language is difficult to understand. To improve the text's understandability, flow, and readability, I recommend the authors engage with a translator, writing coach, or copyeditor. Even, I can see, the editor highlighted the same concern about the writing quality, however, it hasn't significantly improved.	We appreciate your feedbacks. The whole manuscript is checked carefully and fixed the grammatical issues.	Revised texts are in red color.<br>Page: 1-15
Results<br>The author has written the age at first marriage in lines 180 and 181, but the table indicates the age of the women at first birth, which may confuse the reader. Please correct the terminology.<br>"43% of children's mothers got married before the age of 18 years", the authors wrote again on line 181, but the table author has taken the children age groups but do not specify in the above statement.<br>In line number 188, the authors repeated the findings from line number 182, which are related to the 45.8% of births to mothers under the age of 18 years. The author has stated "obesity is lower among women who do not marry at a young age compared to their counterparts" on line 189, however the author's unit of analysis is children, therefore please correct this.<br>There are numerous typos in Table 1, so kindly update these names.<br>Another observation is that the age of the child in month variables labels are not properly specified, such as 36-47 near interval, and the last label started again 47-59; please explain why.<br>The author wrote that the COR for fever is 1.05 in line 249, however, the table shows that it is 1.12.<br>There is an incomplete sentence in line 253 of the manuscript.	Thank you very much.<br>We correct the terminology.<br>We revise the sentence in line 181.<br>We remove the sentence of line 188-189 because of repetition.<br>We revised the sentence on line 189.<br>Thanks. It was a typo and it would be 48-59 instead of 47-59.<br>We also correct the typo in line 249. It would be 1.12.	Revised texts are in red color.<br>Page: 6, 7-12

The authors have written that the OR is 0.79 and that the 20% more likely is shown in line 256; please correct this sentence.<br>The authors used early married women in line 260, but married before their 18 years’ age in line 255, which would confuse the reader. Please use the same variables names that are used in the tables.<br>Some general comments regarding figures and graphs<br>Figure 1 is not a standard graph; please make a standard line graph.<br>Table 2: The P-value was misdefined by the authors.<br>Table 3: The author applied the Omnibus test, however the methodology section did not mention it.	Thanks. We revised the sentence in line 253.<br>We modify the sentence in line 256.<br>We revise this sentences in line 255 and 260.<br>We modify the Figure 1 in the revised manuscript.<br>The p-value presented in Table 2 are correct. However, in texts it was in “<” format. We revise the texts.<br>The methodology section is revised.	
Discussion<br>"Children of women married as a child were significantly more vulnerable to diarrhea and fever than children born to married women as adults," the authors wrote in line 288, I assume that they were referring to the adjusted odds ratio. However, the result in table no 3 is completely different.<br>The author has written in lines 303, 304, and 305 that this research showed that infant, neonatal, post neonatal, and under -5 years of child mortality in Bangladesh increased from 1993 to 2014 over the years and the rate is diminishing steadily over time, yet these are unclear and perplexing.<br>The author writes in line 311 that "a substantial number of social-demographic variables have a significant effect on child married in Bangladesh," however I don't find any evidence that the authors looked into child marriage or that the author's unit of analysis were child married women.<br>The last paragraph of the discussion, in lines 328-329, is not the authors' results, although they do not provide any references.<br>I probably stick to a simple structure and using precise terms. It is necessary to improve the discussion. The authors are confused by the unit of analysis in general throughout the article. Many grammatical errors remain.	Thank you very much for highlighted these points.<br><br>We revised the discussion section as per comments.	Revised texts are in red color.<br>Page: 12-14

In conclusion, the revised version of the manuscript has been produced as per the review outcomes. So, we hope that you will be happy to see this greatly improved version. Once again, we would like to thank you all for your dedication, professional services and cooperation.
