# Supplementary material for: Child marriage and its association with morbidity and mortality of under-5 years old children in Bangladesh
Source: PLoS One. 2022 Feb 9;17(2):e0262927. doi: 10.1371/journal.pone.0262927 (PMC8827428; doi:10.1371/journal.pone.0262927)
Supplement: Attachment — Submitted filename: Response to Reviewers.pdf [file pone.0262927.s005.pdf]

Author responses to the review comments:

We again sincerely thank the Academic Editor and anonymous reviewers for their valuable comments. We have considered all comments and then thoroughly revised and formatted the manuscript. A detailed response to each comment is provided in the following table.

**Editor/Academic Editor comments:**	**Response**	**Note**
Reviewers still not pleased with the revisions. Considering their opinion and my own reading, I am recommending a major revision for this manuscript. This will be a last chance for you to incorporate the reviewers suggestions.<br>Please include the following items when submitting your revised manuscript:<br>A rebuttal letter that responds to each point raised by the academic editor and reviewer(s). You should upload this letter as a separate file labeled 'Response to Reviewers'.<br>A marked-up copy of your manuscript that highlights changes made to the original version. You should upload this as a separate file labeled 'Revised Manuscript with Track Changes'.<br>An unmarked version of your revised paper without tracked changes. You should upload this as a separate file labeled 'Manuscript'.	Thank you very much for providing another chance and valuable comment and feedback to improve the quality as well as suitability for publication of our manuscript.<br>All necessary files are uploaded to the journal system.	All revised texts are in "red" color.
**Reviewer 1 comments:**	**Response**	**Note**
The authors have carried out the revision as suggested and that has improved the manuscript to an extent. I still have some doubts and suggestions regarding the manuscript, which are as follows:<br>1. The image in figure 1 does not seem to be matching with the text in the manuscript. The authors can please clarify this. For ex: in text, it was written "Moreover, the infant mortality rate was 88 in 1993, then gradually decreased to 38 per 1,000 live births over the next two decades. " However, the figure is showing different numbers. Please confirm, if I am wrong in analyzing the figure. A similar mismatch is there in the text at other places too for figure 1.	Thank you very much for carefully checking the manuscript. Actually, the Y-axis level was wrong in Figure 1. Now, Figure 1 is corrected.	All revised texts are in "red" color.<br><br>Page: 9
2. For table 2, if authors could segregate the prevalence of diarrhea by age of the children, it would present a clear picture. Studies have noted the age of the children as an important factor in the prevalence of diarrhea. so presenting diarrhea among children of various ages by women's age at marriage.	Thank you very much. According to your suggestion, we revise the manuscript and add a figure (Figure 2) for presenting the prevalence of diarrhea among children of various ages by women's age at marriage.	Revised texts are in red color.<br>Page: 10-11
3. Please confirm that for table 3, what is the reference category? In table 2, authors have taken two categories for married women - women who married below 18 years and women who married at 18 and above 18 years. But in table 3, authors have	Thanks. We revise the texts.	The revised texts are in red color. Page: 12

taken women as above 18 years old, thereby leaving women who were 18 years old. Why so discrepancy?		
4. Table 1 is providing the differences by age at marriage of the women. I would also suggest to add a table (may be as supplementary) that provides within group differences. For ex: it would be interesting to see the differences between sex, religion, and other covariates. currently, the total adds up for women age grouyp. I want that to be added for covariate.	Thanks. We add a Supplementary Table (S1 Table) in the revised manuscript.	The revised texts are in red color.<br>S1 Table
**Reviewer 3 comments:**	**Response**	**Note**
There have been various necessary changes which are praiseworthy. However, a little English revision still needs to be done. After which the paper is good to publish.<br>Some of the places where revision is needed-<br>Line 46 which in turn affecting<br>Line 49 marriages has happened<br>Line 110 there is necessary – there is a necessity<br>Line 156-They are a – should be –'which are'<br>Line 160- The bivariate and multivariate - Bivariate and multivariate<br>Line 165- child marriage and morbidity and mortality- child marriage, morbidity and mortality<br>178- variable that assign- Variable that assigns	Thank you very much again for carefully checking the manuscript and your feedback. It will help to improve the quality of the manuscript.	Revised texts are in red color.<br>Page: 1, 7, 9, 10
Paper should be thoroughly read and other errors should be rectified to standard English.<br>Usage of article "The" in the sentences should be checked and only put where ever necessary.	Thanks. We have checked and fixed the grammatical issues. Article "The" has been removed from some places.	Revised texts are in red color.<br>Page: 2-16
Mother's BMI has been divided in to- Thin, Normal and Obese, does this include overweight and obese too? There should be some mention of how BMI is categorised.	Thanks, we appreciate your concern. In Table 1, we add the range of BMI for making categories.	Revised texts are in red color.<br>Page: 8
**Reviewer 4 comments:**	**Response**	**Note**
I would like to make a couple of minor suggestions. They are listed below:<br>The authors have incorporated the most of the previous comments into the manuscript.<br>But, some additional comments authors should be considered.<br>Introduction<br>In line numbers 52 and 53, the authors describe the prevalence of child marriage in the Dominican Republic and Brazil, how it is justified, and whether or not both countries have the same socioeconomic conditions as Bangladesh. It would be better to compare the Dominican Republic and Brazil to Pakistan and Indonesia, where the	Thank you very much for your appreciation and insightful feedback. We believe that your comments help to improve the quality of the manuscript.<br><br>We revise the manuscript considering the information of Muslim countries like Pakistan, Indonesia, and Afghanistan.<br><br>We add the BMI range for the "thin" group in line 89.	Revised texts are in red color.<br>Page: 2, 3,

majority of the population adheres to the Muslim religion.<br>Line 89, the authors have written the putting them in the thin group, what is the thin group, the sentence is unclear.		
Results<br>Still, Figure is not a conventional graph; please see the other published paper graphs.<br>In the result section, the authors have written that the COR but written Adjusted AOR, the author can write same AOR instead of adjusted AOR.	Thanks for carefully checking the manuscript. We replace Figure 1 and incorporate all your suggestions in the revised version of the manuscript.	Revised texts are in red color.<br>Page: 9, 11-13

In conclusion, the revised version of the manuscript has been produced as per the review outcomes. So, we hope that you will be happy to see this greatly improved version. Once again, we would like to thank you all for your dedication, professional services, and cooperation.
