# Supplementary material for: Child marriage and its association with morbidity and mortality of under-5 years old children in Bangladesh
Source: PLoS One. 2022 Feb 9;17(2):e0262927. doi: 10.1371/journal.pone.0262927 (PMC8827428; doi:10.1371/journal.pone.0262927)
Supplement: Attachment — Submitted filename: Response to Reviewers.pdf [file pone.0262927.s006.pdf]

Author responses to the review comments:

We further sincerely thank the Academic Editor for his careful checking and providing feedback and comments. We have considered all comments and then thoroughly revised and formatted the manuscript. A detailed response to each comment is provided in the following table.

Editor/Academic Editor comments:	Response	Note
I am sending back the paper to you for a minor revision. Please provide the full regression tables in the supplementary file and attach with the paper. Also, in predictors variables, your using both 'socio-economic and demographic' factors but why you have written only 'demographic factors' in column heading of the Tables. The logistic regression equation is looks like copy paste from the reference books. Can you modify the equation by placing the "Y" variable name, and the key Predictor (X) name in the equation. For example, Y(child health) = alpha + beta.x(child marriage).........xi+error term. Also, please read the paper for language errors once again. Although, I am recommending this paper because it is about child marriage, but the analyses are not very impressive to me.<br><br>Please include the following items when submitting your revised manuscript:<br>A rebuttal letter that responds to each point raised by the academic editor and reviewer(s). You should upload this letter as a separate file labeled 'Response to Reviewers'.<br>A marked-up copy of your manuscript that highlights changes made to the original version. You should upload this as a separate file labeled 'Revised Manuscript with Track Changes'.<br>An unmarked version of your revised paper without tracked changes. You should upload this as a separate file labeled 'Manuscript'.	Thank you very much for providing another chance and valuable comment and feedback to improve the quality as well as suitability for publication of our manuscript.<br><br>We add a supplementary Table (S2 Table) to summarize the results of logistic regression.<br><br>We replace "demographic factors" by "Socio-economic and demographic characteristics" in Tables.<br><br>The equation of logistic regression is revised as per feedback.<br><br>All necessary files are uploaded to the journal system.	All revised texts are in "red" color.<br>Page: 6-8, 13, S1 Table, S2 Table

In conclusion, the revised version of the manuscript has been produced as per the review outcomes. So, we hope that you will be happy to see this greatly improved version. Once again, we would like to thank you all for your dedication, professional services, and cooperation.
