# Supplementary material for: Child marriage and its association with morbidity and mortality of under-5 years old children in Bangladesh
Source: PLoS One. 2022 Feb 9;17(2):e0262927. doi: 10.1371/journal.pone.0262927 (PMC8827428; doi:10.1371/journal.pone.0262927)
Supplement: Attachment — Submitted filename: Response to Reviewers.docx [file pone.0262927.s003.docx]

**Author responses to the review comments:**

We would like to sincerely thank the Academic Editor for your valuable comments. We have considered all comments and then thoroughly revised and formatted the manuscript. A detailed response to each particular comment is provided in the tables as follows.

| **Editor/Academic Editor comments:** | **Response** | **Note** |
| --- | --- | --- |
| Although the research question of this study is very important, but in its current form it doesn't suits for publication in PLOS One. The paper is less likely receive favourable opinion from the reviewers. The analytical plan and tools are very weak and doesn't support the research question. Thus, I recommend authors to revise and resubmit to PLOS One or any other journal. Moreover, dont attempt to measure impact or causation from a cross-sectional data. Try to use PSM to address the adverse affect of child marriages on child health outcomes. Consult following papers for methods, concept and framework of analyses.  Vikram, Kriti. "Early marriage and health among women at midlife: Evidence from India." Journal of Marriage and Family (2021).  Goli S, Rammohan A, Singh D. The Effect of Early Marriages and Early Childbearing on Women's Nutritional Status in India. Matern Child Health J. 2015 Aug;19(8):1864-80. doi: 10.1007/s10995-015-1700-7. PMID: 25656721.  Goli, S. (2016). Eliminating child marriages in India: Progress and prospects. Child Rights Focus-Action Aid. https://www.actionaidindia.org/publications/eliminating-child-marriage-in-india/ | Thank you very much for your valuable comment and feedback. We believe that it will be helpful to improve the quality of the manuscript.  We have cited the suggested papers in our manuscript.  Thanks for your suggesting to use PSM for finding the adverse effect of child marriages on child health outcomes. However, in light of the existing literature, it is found that there are several manuscript used the logistic regression to identify the association between child marriage and child health outcome. Therefore, we think this would be a potential topic for future research. | All revised texts are in “red” color.  Page 1, 4, 7-9, 14-15, 18-19 |
| When submitting your revision, we need you to address these additional requirements.  1. Please ensure that your manuscript meets PLOS ONE's style requirements, including those for file naming. The PLOS ONE style templates can be found at  https://journals.plos.org/plosone/s/file?id=wjVg/PLOSOne_formatting_sample_main_body.pdf and  https://journals.plos.org/plosone/s/file?id=ba62/PLOSOne_formatting_sample_title_authors_affiliations.pdf | Thanks. We ensure you that we follow the journal guidelines for formatting purpose. |  |
| 2. We suggest you thoroughly copyedit your manuscript for language usage, spelling, and grammar. If you do not know anyone who can help you do this, you may wish to consider employing a professional scientific editing service.  Whilst you may use any professional scientific editing service of your choice, PLOS has partnered with both American Journal Experts (AJE) and Editage to provide discounted services to PLOS authors. Both organizations have experience helping authors meet PLOS guidelines and can provide language editing, translation, manuscript formatting, and figure formatting to ensure your manuscript meets our submission guidelines. To take advantage of our partnership with AJE, visit the AJE website (http://learn.aje.com/plos/) for a 15% discount off AJE services. To take advantage of our partnership with Editage, visit the Editage website (www.editage.com) and enter referral code PLOSEDIT for a 15% discount off Editage services. If the PLOS editorial team finds any language issues in text that either AJE or Editage has edited, the service provider will re-edit the text for free.  Upon resubmission, please provide the following:  The name of the colleague or the details of the professional service that edited your manuscript  A copy of your manuscript showing your changes by either highlighting them or using track changes (uploaded as a *supporting information* file)  A clean copy of the edited manuscript (uploaded as the new *manuscript* file) | Thank you very much for your valuable comment and suggestions that help improve the manuscript's quality. We thoroughly checked the grammatical issues and also check by Grammarly software.  We upload the required files following the guidelines provided. | All revised texts are in “red” color.  Page 1-15 |
| 3. Thank you for stating the following financial disclosure:  “No. The funders had no role in study design, data collection and analysis, decision to publish, or preparation of the manuscript.”  At this time, please address the following queries:  a) Please clarify the sources of funding (financial or material support) for your study. List the grants or organizations that supported your study, including funding received from your institution.  b) State what role the funders took in the study. If the funders had no role in your study, please state: “The funders had no role in study design, data collection and analysis, decision to publish, or preparation of the manuscript.”  c) If any authors received a salary from any of your funders, please state which authors and which funders.  d) If you did not receive any funding for this study, please state: “The authors received no specific funding for this work.”  Please include your amended statements within your cover letter; we will change the online submission form on your behalf. | Thank you very much.  The authors received no specific funding for this work.  We add this statement in the manuscript and cover letter. | Revised texts are in red color.  Page: 15 |
| 4. Your ethics statement should only appear in the Methods section of your manuscript. If your ethics statement is written in any section besides the Methods, please move it to the Methods section and delete it from any other section. Please ensure that your ethics statement is included in your manuscript, as the ethics statement entered into the online submission form will not be published alongside your manuscript. | Thanks. We move the ethical statement from the end of the manuscript to the Methods section. | The revised texts are in red color. Page: 5 |
| 5. Please ensure that you refer to Figure 2 in your text as, if accepted, production will need this reference to link the reader to the figure. | Thanks. We have removed Figure 2 from our manuscript. | Page: 12 |
| 6. We note that Figure 2 in your submission contain map images which may be copyrighted. All PLOS content is published under the Creative Commons Attribution License (CC BY 4.0), which means that the manuscript, images, and Supporting Information files will be freely available online, and any third party is permitted to access, download, copy, distribute, and use these materials in any way, even commercially, with proper attribution. For these reasons, we cannot publish previously copyrighted maps or satellite images created using proprietary data, such as Google software (Google Maps, Street View, and Earth). For more information, see our copyright guidelines: http://journals.plos.org/plosone/s/licenses-and-copyright.  We require you to either (1) present written permission from the copyright holder to publish these figures specifically under the CC BY 4.0 license, or (2) remove the figures from your submission:  a. You may seek permission from the original copyright holder of Figure 2 to publish the content specifically under the CC BY 4.0 license.  We recommend that you contact the original copyright holder with the Content Permission Form (http://journals.plos.org/plosone/s/file?id=7c09/content-permission-form.pdf) and the following text:  “I request permission for the open-access journal PLOS ONE to publish XXX under the Creative Commons Attribution License (CCAL) CC BY 4.0 (http://creativecommons.org/licenses/by/4.0/). Please be aware that this license allows unrestricted use and distribution, even commercially, by third parties. Please reply and provide explicit written permission to publish XXX under a CC BY license and complete the attached form.”  Please upload the completed Content Permission Form or other proof of granted permissions as an "Other" file with your submission.  In the figure caption of the copyrighted figure, please include the following text: “Reprinted from [ref] under a CC BY license, with permission from [name of publisher], original copyright [original copyright year].”  b. If you are unable to obtain permission from the original copyright holder to publish these figures under the CC BY 4.0 license or if the copyright holder’s requirements are incompatible with the CC BY 4.0 license, please either i) remove the figure or ii) supply a replacement figure that complies with the CC BY 4.0 license. Please check copyright information on all replacement figures and update the figure caption with source information. If applicable, please specify in the figure caption text when a figure is similar but not identical to the original image and is therefore for illustrative purposes only.  The following resources for replacing copyrighted map figures may be helpful:  USGS National Map Viewer (public domain): http://viewer.nationalmap.gov/viewer/  The Gateway to Astronaut Photography of Earth (public domain): http://eol.jsc.nasa.gov/sseop/clickmap/  Maps at the CIA (public domain): https://www.cia.gov/library/publications/the-world-factbook/index.html and https://www.cia.gov/library/publications/cia-maps-publications/index.html  NASA Earth Observatory (public domain): http://earthobservatory.nasa.gov/  Landsat: http://landsat.visibleearth.nasa.gov/  USGS EROS (Earth Resources Observatory and Science (EROS) Center) (public domain): http://eros.usgs.gov/#  Natural Earth (public domain): http://www.naturalearthdata.com/ | Thank you very much. We remove Figure 2 from our manuscript. | Page: 12 |

In conclusion, the revised version of the manuscript has been produced as per the review outcomes. So, we hope that you would be happy to see this greatly improved version. Once again, we would like to thank you all for your dedication, professional services and cooperation.
